# Acculturation and Nutritional Health of Immigrants in Canada: A Scoping Review

**DOI:** 10.1007/s10903-013-9823-7

**Published:** 2013-04-18

**Authors:** Dia Sanou, Erin O’Reilly, Ismael Ngnie-Teta, Malek Batal, Nathalie Mondain, Caroline Andrew, Bruce K. Newbold, Ivy L. Bourgeault

**Affiliations:** 1 Interdisciplinary School of Health Sciences, Faculty of Health Sciences, University of Ottawa, Thompson Hall—35 University Private (room 036), Ottawa, ON K1N 6N5 Canada; 2 Nutrition Program, Faculty of Health Sciences, University of Ottawa, Ottawa, ON Canada; 3 United Nations Children’s Fund (UNICEF), Pétion-Ville, Haiti; 4 Department of Sociology and Anthropology, Faculty of Social Sciences, University of Ottawa, Ottawa, ON Canada; 5 School of Political Sciences, Faculty of Health Sciences, University of Ottawa, Ottawa, ON Canada; 6 School of Geography and Earth Sciences, Faculty of Sciences, McMaster University, Hamilton, ON Canada

**Keywords:** Dietary acculturation, Immigrant health, Canada, Nutrition

## Abstract

Although recent immigrants to Canada are healthier than Canadian born (i.e., the *Healthy Immigrant Effect*), they experience a deterioration in their health status which is partly due to transitions in dietary habits. Since pathways to these transitions are under-documented, this scoping review aims to identify knowledge gaps and research priorities related to immigrant nutritional health. A total of 49 articles were retrieved and reviewed using electronic databases and a stakeholder consultation was undertaken to consolidate findings. Overall, research tends to confirm the Healthy Immigrant Effect and suggests that significant knowledge gaps in nutritional health persist, thereby creating a barrier to the advancement of health promotion and the achievement of maximum health equity. Five research priorities were identified including (1) risks and benefits associated with traditional/ethnic foods; (2) access and outreach to immigrants; (3) mechanisms and coping strategies for food security; (4) mechanisms of food choice in immigrant families; and (5) health promotion strategies that work for immigrant populations.

**Electronic supplementary material:**

The online version of this article (doi:10.1007/s10903-013-9823-7) contains supplementary material, which is available to authorized users.

## Introduction

International immigration has been identified by policy-makers as a critical strategy for Canada’s economic development and the most important component of the country’s population growth. Foreign-born Canadians were estimated to be 13 million people in 2006 (19.1 % of the general population) and it is anticipated that the country will continue to receive more than 200,000 immigrants annually, accounting for 60 % of its annual population growth [1].

Upon arrival, Canadian immigrants usually have fewer chronic conditions compared to the native-born population [2–5]. This trend is often referred to as the *Healthy Immigrant Effect* and has been well documented in the Canadian literature [4–9] and around the world [10–16]. The “Healthy Immigrant Effect” refers to the fact that immigrants tend to be healthier than the Canadian-born population when they first arrive in the country due to the selection effect of immigrants. Indeed, in order to immigrate, candidates for immigration undergo medical screening and people who have health problems generally do not immigrate. However, immigrants tend to experience a rapid deterioration in their general health status after settlement in Canada due to lifestyle changes including patterns of physical activity and dietary habits [10–17].

Among the many factors that contribute to the loss of the *Healthy Immigrant Effect* is nutrition transition. This transition is thought to be mediated by *dietary acculturation*, which is the process by which immigrants adopt the dietary practices of the host country [18]. Researchers in the USA and Europe have found positive associations between the level of dietary acculturation and risk factors for chronic diseases [10, 14–16, 19]. Further, immigration related cultural changes have been found to be independently associated with high blood pressure [11, 14]. The impact of acculturation can be more significant than changes in diet or physical activity [14], and may increase the risk of obesity across generations [13]. Although there is much evidence supporting the existence of the loss of the Healthy Immigrant Effect in Canada, little is known about immigrant nutritional health and immigration related dietary changes. Additionally, it is unclear under which circumstances immigrants to Canada undergo dietary acculturation and to what extent nutrition and health transitions are affected by acculturation. It is important to acknowledge that newcomers can acculturate into the host culture while retaining or not retaining their cultural roots [12]. Therefore, the impact of acculturation on health may depend on the path and the degree to which immigrants maintain their former cultural identity as well as on the extent to which they adopt the cultural practices of their new homeland [12]. In any case, the lack of contextual information impedes the development of appropriate health promotion practices, interventions and policies that could help mitigate this problem.

This scoping review was undertaken to: (1) identify the extent and the nature of research activities related to the nutritional health of immigrants to Canada; (2) examine potential relationships between acculturation measures, dietary behaviours and selected corresponding health conditions; and (3) identify potential gaps in the existing literature and key research priorities that will better inform practice and advance health policies. Understanding the relationships between acculturation, dietary behaviours and health transitions is the first step towards the development of appropriate intervention strategies for a multicultural population that requires more and more cultural competencies.

## Methods

For the purpose of this study, we conducted a scoping review of national, provincial and local studies that focus on the nutritional health of immigrants living in Canada. A scoping review is an increasingly utilized approach for reviewing health research evidence in order to convey the breadth and depth of a field [20, 21]. Scoping reviews are thought to be particularly appropriate in areas with emerging evidence in which the paucity of data makes it difficult to undertake systematic reviews [21, 22]. Such methodology offers an opportunity to examine the extent and nature of research related to immigrant nutritional health by incorporating a range of studies with different designs (qualitative, quantitative or mixed) and from both peer-reviewed and grey literature regardless of the quality of the study. Arksey and O’Malley [22] outline a six-step methodological framework for conducting scoping reviews including (1) identifying the research question; (2) identifying relevant studies; (3) carefully selecting studies; (4) charting the data; (5) collating, summarizing and reporting the results; and (6) conducting a consultation exercise with consumers and stakeholders.

### Identification of the Research Question

The first task for the research team was to identify the research question and subsequent study objectives. Thereafter, the keywords to be used for the literature search and the inclusion criteria were defined.

### Identification of Relevant Articles

In the current study, papers were identified using three electronic databases; namely Embase, Medline and Web of Science. Backward from reference lists and key review journal and hand searching from Google were also used using the same key words. The following keywords were used for the search:

- Canad*^1^
- AND (immigra* OR acculturation OR migra*)
- AND (nutrition* OR diet* OR food*OR eating OR obesity OR overweight OR diabet* OR cardiovascular disease)

**Fig. 1:**
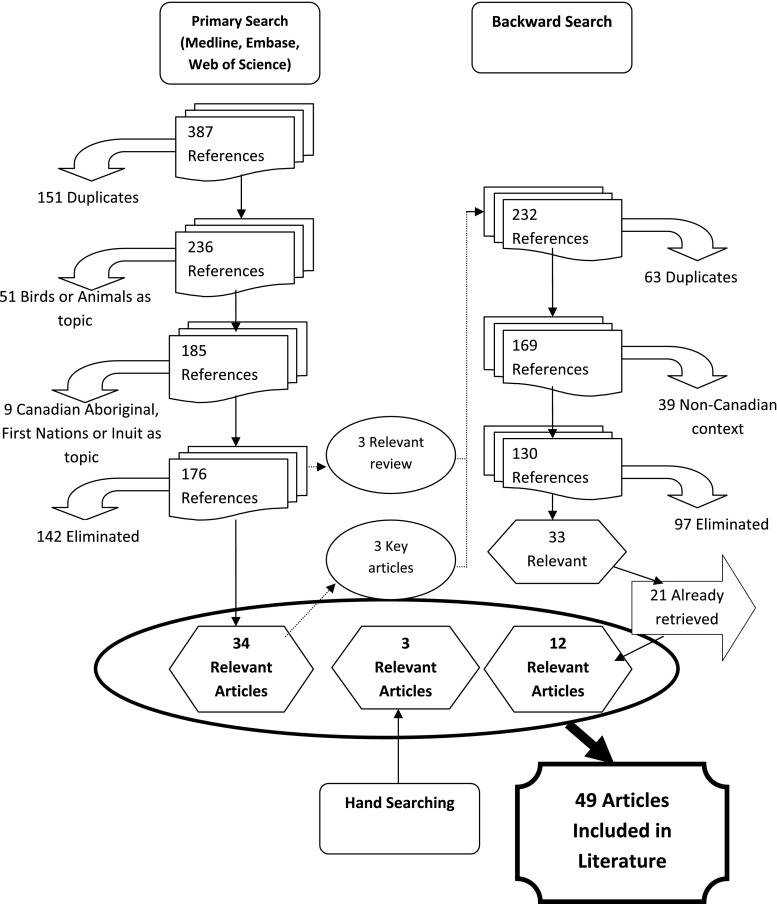
Flowchart of the article retrieval process

The search was limited to articles published between 1990 and 2010 written in English or French. Figure 1 summarizes the process used to identify and select papers included in this review. The inclusion criteria were:

- Original research paper;
- At least partly based on Canadian data (most were fully based on Canadian data);
- Study focused mainly on or addressed diet, food, nutrition and/or related health issues such as overweight/obesity, diabetes and cardiovascular disease.

The following documents were not included in the review: conference and poster abstracts, letters, commentaries, editorials, as well as general reviews, meta-analysis studies, and practice guideline papers. Qualitative studies that did not mention risk factors or prevalence were also excluded.

### Study Selection

The initial database search yielded a total of 387 articles. Using Refworks to check for close matches or duplicated articles, 151 duplicates were removed. Fifty-one [51] additional articles were removed because they were focused on non-human subjects (i.e. bird or animal migration patterns). Another nine were deleted because they were focused on Canadian Aboriginal populations. Three review articles and three of the selected articles were used for backward reference searching (i.e. by reviewing the references of the selected articles). The 232 references obtained by this method were entered into a RefWorks folder and screened using the same exclusion criteria as for the primary search. This process yielded 12 additional articles that were relevant for the literature review. Finally, we completed a manual search through a general internet browser (Google Scholar) and using the same criteria as above.

### Charting the Data

A total of 49 articles were retrieved and included in the literature review. For the purpose of this review, we focused primarily on variables relevant to acculturation, food and diet and/or nutrition. Each of the 49 articles was read thoroughly and all pertinent information was extracted The data recorded from each article included the study design, the study population and sample size, measurements of diet/nutrition and/or health, indicators of acculturation, major findings and policy implications. This information was then entered onto a charting form using Microsoft Excel by one of the study co-authors (EO) and double checked by a second co-author (DS).

### Summarizing and Reporting the Results

The information extracted from the 49 articles was analyzed by three of the co-authors (EO, DS, and NTI) using a narrative synthesis (as opposed to statistical) of the findings. Key themes related to measurements of acculturation, dietary behaviours; nutrition and health transitions, including factors contributing to these transitions, were reported and discussed.

### Stakeholder Consultation

A stakeholder consultation occurred midway through the review and literature extraction process. It consisted of a one-day symposium held in Ottawa, which was entitled “*Immigration, Dietary Acculturation and the Health of Neo*-*Canadians: State of Knowledge, Dynamics and Research Priorities*”.

Participants included researchers, designated members of immigrant cultural associations and representatives from governmental agencies, nutrition related professional organizations, community health centres and organizations working for immigrant settlement. The symposium was preceded by a survey that was completed by 16 of the participating stakeholders including community health centers, immigrant organizations, governmental agencies and non-governmental organizations working in the area of immigrant health and nutrition. In total, 46 individuals affiliated with 30 organizations whose mandate touches immigrant’s health and nutrition across Canada attended the symposium.

The symposium began with presentations on the *Healthy Immigrant Effect* and Health Canada Initiatives in Promoting Healthy Eating in Multicultural Settings. Thereafter, findings from the pre-symposium survey completed by over 16 organizations were used to complement the major findings from the literature review and were presented as a backgrounder for discussion. The symposium was facilitated using an electronic meeting system (EMS) by an expert facilitator with no prior link to the research team. The Queen’s University EMS, called “the Decision Centre” is an innovative facilitation process developed at the Queen’s School of Business that combines expert facilitation with a state of the art group decision support system to enable groups to rapidly accelerate idea generation and consensus building. In small groups, participants brainstormed over 35 knowledge gaps that were then used to inform research areas. Over 20 research areas were identified in connection with these knowledge gaps and were then clustered into major categories by merging the sub topics. Specific research questions, strategies for increasing immigrant participation in research and relevant stakeholders were later discussed in relation to each area.

## Results

### Characteristics of the Studies

A total of 49 peer-reviewed articles and government publications meeting all of the inclusion and exclusion criteria were included in the literature review. Twenty-eight [28] studies were based on national surveys and 15 of these were based on secondary data analysis from various cycles of the national surveys starting in 1994. Of the remaining studies, eight were conducted in Ontario, seven in Québec, four in British Columbia, one in Newfoundland and one in both Québec and Ontario. Among these 21 studies, 16 were focused in large metropolitan cities including Montréal, Toronto and Vancouver.

### Study Populations

Most of the studies involved adolescents and/or young adults to middle-age populations. Five studies considered only older adults aged over 52 years; one focused on pregnant women and only one included school children aged 4–13 years. Nearly all of the studies considered first-generation immigrants exclusively and only one study addressed the dimension of intergenerational impact on health outcomes for the Canadian-born children of immigrants.

With the exception of the studies based on the Ontario Health Survey, all of the studies that were not based on large national databases targeted particular ethnic subgroups comprising a maximum of a few hundred participants. Five studies involved Chinese immigrants, three involved Haitian immigrants and three were focused on South Asian immigrants. Other subgroups targeted by individual studies included South-East Asians, Francophone West Africans, Lebanese, Filipinos, and Europeans. Eight studies focused exclusively on women while only one was focused exclusively on males.

For the most part, immigrants were classified only by their country of origin or as an immigrant/non-immigrant. Only one study directly considered immigrants by their status as a refugee, economic or familial class immigrant [23]. This particular study concluded that immigrant status affects the likelihood of health transition.

### Measurement of Acculturation

The most common dimension of acculturation captured in the studies was the duration of stay in Canada, which was the primary measure of acculturation in 33 out of the 49 studies considered for this review. Other frequently employed measures included birthplace, country of origin, age at arrival to Canada and language use or language proficiency. Author-specific scales of acculturation were utilized less often but were applied in a few of the studies. These specific indicators included ethnic identification, level of social interaction within the ethnic community, cultural knowledge, religious affiliation, social marginalization, visible minority status or race, family structure and gender roles and adherence to traditional health beliefs.

### Measurements of Diet and Health

Dietary patterns were evaluated in approximately half of the studies. The most common tools used for dietary assessment were food frequency questionnaires and 24-h recalls of food consumption, which were applied in 13 of the studies reviewed. A number of studies used semi-structured questionnaires and/or interviews alone or in conjunction with a healthfulness score for diet, food choice motives and the pre-immigration and post-immigration consumption level of fruits, vegetables and various fats.

The concept of a dietary acculturation scale was used by some authors. For example, scores on Chinese and American dietary behaviour scales were used to assess the level of acculturation undergone by Chinese living in Vancouver BC [24, 25]. This model was also applied in a subsequent study that focused on the dietary acculturation of Africans in Montréal [26]. A *dietary transition model* was applied to categorize the diets of Haitians in Montréal into four broad groups: *Traditional*, *Pre*-*Western*, *Western* and *Modern* [27].

Most studies included a measurement of self-assessed health status or perceived change in health status and frequency of health care service utilization. Other variables commonly used included the presence of chronic disease, BMI status and/or health related behaviours (smoking, alcohol consumption, level of physical activity and stress). Mental health issues were investigated in only one study [28].

### Acculturation and Health Transition

A key indicator of acculturation, time since immigration, was correlated with an increased BMI in the majority of the studies reviewed. In general, the prevalence of obesity was found to be higher among native-born Canadians compared to non-white immigrants, however, the prevalence of obesity among immigrants was found to increase with their duration of stay in Canada. These findings were consistent with other health markers including declining self-assessed health, increased frequency of health care usage and onset of chronic conditions. In one study, however, the frequency of doctor or hospital visits among immigrants was found to be equal to that of the native-born population regardless of time since immigration [23]. It is also noteworthy that immigrants were found to be generally less active than non-immigrants [7]. Concerns about thinness typical of Western body ideals were shown to increase with years since immigration among immigrant women [28] and adherence to traditional health beliefs declined with a younger age at immigration after 10 years of residency in Canada [29].

Overall, individual studies tend to confirm the *Healthy Immigrant Effect* with recent immigrants being in better health (lower BMI, better SAH, and lower prevalence of chronic conditions) compared to immigrants having moved to Canada earlier and non-immigrants regardless of their socioeconomic status. Among the studies that focused on immigrant health, there is heterogeneity in the results as some studies provided mixed support or failed to confirm the *Healthy Immigrant Effect* [6, 30, 31]. European immigrants were the most likely to report poor health status, chronic conditions and hospitalization [5, 31] and increased BMI [4], independent of time since immigration. While European and American immigrants tended to represent a greater proportion of long-term immigrants to Canada in 1957 (95 %), this trend has shifted to only 29 % in 1990 [32].

### Acculturation and Dietary Transition

Canada’s *Food Guide* emphasizes the need for a diverse and balanced diet. Studies that investigated nutrient intakes concluded that, in general, immigrants (particularly those of Asian origin) are at higher risk of inadequate intake of calcium, iron and protein [33]. In spite of this, long exposure to Canadian culture tends to be associated with an increased fat and sodium intake [23, 34]. One study involving older adult immigrants in London, Ontario found that continuance of traditional eating habits and consumption of special ethnic foods can increase the risk of poor nutrition, particularly sodium intake, which was found to be as high as 238–474 % of the daily recommended intake for some individuals [35]. Overall carbohydrate intake tended to be lower among immigrants [36]. Positive diet behaviours with regard to fruit and vegetable intakes were also reported among Chinese women in living Vancouver [25, 34].

Consistently across studies, most ethnic groups try to maintain their traditional diet to some degree, which is believed to be healthier. Francophone Africans in Montréal tend to maintain their home country’s dietary habits independently of time since migration [37]. Punjabi women residing in West Toronto maintain a traditional diet that is generally considered to be healthier (plant-based, high in protein and complex carbohydrates) compared to a typical Western diet [38]. One study found that among Italian and Greek immigrants residing in a major metropolitan area of eastern Canada, *ethnic identity* was positively correlated with traditional food consumption, and negatively related to the consumption of Western convenience foods, whereas the concept of *acculturation* had no effect on immigrant food choice [39]. In contrast to these results, some authors found that Haitian immigrants easily undergo dietary transitions from their healthier traditional diet to a more Western diet [27, 37].

### Factors Contributing to Dietary Transition

Pillarella et al. [26] found that at least some changes in dietary habits after settlement in Canada is largely inevitable. The extent of these changes was dependent on several factors such as economic status, country of origin and living environment within Canada. Enabling factors for maintaining traditional dietary habits among Francophone West African immigrants in Montréal included learned cooking skills, taste preferences and health knowledge while drivers of dietary acculturation included time constraints, the influence of new interpersonal relationships, unfamiliarity with grocery procurement patterns and Canadian nutrition discourse.

Lack of knowledge regarding nutrition information provided by the Canadian government and unfamiliarity with Canadian foods and cooking techniques were reported as factors contributing to the reluctance of Chinese and Indian immigrant women to prepare Western foods [18, 40, 41]. Changes due to living environment, availability of foods, and lifestyle factors such as urbanization and language barriers have also been shown to impede the Punjabi women’s efforts to maintain their traditional dietary practices. Lack of time for food preparation and unavailability of traditional ingredients and equipment in Canada were also identified as barriers to maintaining a traditional diet [35]. Availability and affordability of traditional foods were reported as important barriers to healthy eating among older Punjabi men living in British Columbia who tended to prefer traditional dishes [42].

The socioeconomic status (SES) of immigrants was also shown to play a role in dietary and health transitions among immigrants. Low SES was associated with a transition from traditional to Western diet among Haitian immigrants residing in Montréal [27, 37]. Johnson and Garcia [35] reported that barriers to healthy eating among older adults (aged 59–81) in London, Ontario include a low level of education, poverty and transportation constraints. Immigration related stress was shown to contribute to communication barriers, social isolation and financial insecurity, which may in turn affect the eating behaviors and health status of newcomers [34]. Lear et al. [34] reported a potential inner conflict for immigrants who maintain the values of their home culture while simultaneously adapting to those of their new country. Another study found that long-term immigrants experience more stress and pressure to adhere to host-country cultural norms due to lack of social support [28].

These findings also highlighted the effect that the source country circumstance has on SES status, which may explain some discrepancies in health status between recent and long-term immigrants. For example, psychological stress has been found to be a significant predictor of hypertension among Asian immigrants and this risk factor rises with increased number of years since migration [43].

### Knowledge Gaps and Research Priorities

The pre-symposium survey, which was completed by 16 organizations and researchers, identified a range of knowledge gaps. Knowledge gaps that emerged from the literature review and the pre-symposium survey are summarized in Table 1. The brainstorming during the symposium yielded over 35 knowledge gaps pertaining to two major domains: (1) mechanisms by which immigration related factors affect nutrition and health; and (2) evidence based interventions for improving the nutritional health of immigrants. These gaps were narrowed down through a prioritization process and clustered in six key categories by merging the sub categories. The six categories (themes) were equally distributed among the two major domains of knowledge gaps. The knowledge gaps related to mechanisms of immigrant nutrition and health transitions included: (1) the impact of acculturation on dietary habits and health of Canadian immigrants; (2) immigrant youth’s dietary patterns and the perceptions, magnitude and health impacts of food insecurity among Canadian immigrants and (3) epidemiology of selected micronutrient deficiencies among immigrants (i.e. vitamin D). The unanswered questions with regards to intervention strategies are thought to be linked with: (1) designing nutrition promotion programs that can better meet the needs of immigrants; (2) potential use of a positive deviance model with a focus on particular groups of immigrants who have maintained a healthy diet and, (3) effective nutrition communication tools typical of the Canadian nutrition discourse (i.e. Canada’s Food Guide and Food Labelling).

**Table 1:** Summary of the available evidence and knowledge gaps on nutritional health related issues among Canadian immigrants from the literature review

What we know···	What we don’t know yet···
The healthy immigrant effect (HIE): there is an overall negative effect on the health of immigrants associated with time since migration to Canada	What specifically is the role of diet and acculturation? How does the enculturation (tendency to maintain values and beliefs of the home country)—acculturation dynamic affect the dietary and health transitions among immigrants?
Dietary habits inevitably change after immigration to Canada	How, why and to what extent? How does the exposure to Canadian culture affect the nutrition knowledge, perceptions and beliefs of immigrants? What are their knowledge and perceptions of the host country nutrition discourse?
Some individuals tend to maintain their traditional diet that is often healthier that *Western* diets	What are the challenges in maintaining a traditional diet? How can maintenance be facilitated while enhancing nutrition knowledge and practice? Can we conciliate and/or simultaneously promote healthy traditional foods and healthy western foods?
South East Asians, Caribbeans, Africans and Latinos are at a high risk for nutrition related chronic conditions including diabetes and cardiovascular disease.	What are the age standardized BMI cut-offs that are appropriate for identifying individuals at risk in these groups? What are the dietary and activity behaviours of these mostly understudied groups underlying an increased or reduced risk of chronic conditions? Is there any role for the diet-gene interactions?
Dietary transition undoubtedly contributes to the health transition among Immigrant, but diet is one risk factor among many	How do social determinants affect the relationships between acculturation, immigrant dietary behaviours, and health? What are the specific role of the different levels of determinants: individual (gender, age); behaviours; social (poverty, religion, social support, education, culture), and physical environment (winter, transportation)? What are the most important driving factors to target in nutrition related health promotion? What is the role of physical activity?
There is an increasing prevalence of childhood obesity and adolescent onset of type II diabetes in Canada	What are the dietary risk factors for type II diabetes and other chronic conditions among immigrant children? How do these risk factors vary across generations, immigrant groups (cultural origin and religion) and types (refugees, skilled workers, and family class), gender, and over time?
Availability and accessibility of traditional foods, financial insecurity, and limited transportation are important barriers to healthy eating	What is the magnitude of food insecurity among immigrants? How do immigrants experience and cope with these issues?
There tends to be a consensus that culturally appropriate interventions including promoting traditional foods for immigrants is important to ensure better health equity	What works: what cost effective interventions related to immigrant dietary behaviours, nutrition and health are available for scaling up? What are the nutritional values and potential safety of traditional foods? What is the best way to include traditional foods in health promotion programmes? Which groups should be prioritized for health promotion: women? Children? Refugees?

The five research priorities that arose from the prioritization process included (1) risks and benefits of traditional/ethnic foods; (2) access and outreach to immigrants; (3) mechanisms and coping strategies for food security among immigrant families; (4) mechanisms of food choice in immigrant families; and (5) effective health promotion strategies that work for immigrant populations including health promotion, education and training in nutrition and interventions targeting the broader social determinants of health.

To enhance immigrant participation in nutrition research, participants suggest the following ten strategies:

1. Identifying and involving community champions : peer leaders, community champions and outreach workers
2. Reducing logistical barriers that can hinder immigrant participation in research (transportation, child care···)
3. Providing incentives for taking part in research (monetary benefits, grocery coupons, bus tickets, meal, babysitting···), meals during focus groups discussion and more equitable financial partnerships through compensation for research participants (gifts-in-kind/cash, etc.)
4. Implementing appropriate community needs and assets assessment that will inform knowledge, perceptions and practices within the community
5. Aligning research approaches to cultural norms and practices within the community and potential promising target approach: «One size does NOT fit all»
6. Building trust with community members
7. Involving community members in the research process from planning to dissemination
8. Building research capacity within immigrant communities
9. Enhancing ownership of the research process, data and outcomes
10. Using other miscellaneous strategies such as targeting women and other specific trusted groups among the community members

## Discussion

There is strong evidence that the burden of nutrition related chronic conditions for immigrants steadily increases after settlement in the host country. Despite increasing data on the health transitions among individuals who immigrate to Canada, little is known about specific factors that contribute to these transitions and to what extent these transitions occur. In addition, there are increasing dietary and nutrition transitions leading to chronic disease in developing countries, whose inhabitants are immigrating to Canada. Therefore, as the immigrant population continues to grow, the impact on Canadian health systems is expected to increase. To help mitigate this emerging burden of disease in Canada, evidence based data relevant for both health professionals and policymakers is needed.

This scoping review was undertaken to assess the current literature on issues pertaining to immigrant nutritional health and to identify potential priorities for research. Findings suggest that at least some changes in dietary habits after settlement in Canada are inevitable, and that these changes may cause unhealthy transitions. Despite a recent increase in research related to immigrant health in Canada, important knowledge gaps remain regarding food and nutrition issues. This lack of focus on nutritional health related research involving the Canadian immigrant population is leaving an unfilled information gap and creating a barrier towards maximum health equity. This literature review has identified several gaps and stakeholders have prioritized these gaps and identified the most pressing research needs for health promotion.

While acculturation may increase the risk for obesity and related chronic conditions, it may also protect against some nutritional deficiencies such as calcium, vitamin D and iron. Indeed, immigrants (especially from Asian countries) tend to consume less protein, calcium and iron compared to non-immigrants [33]. Therefore, the risk for conditions such as osteoporosis and iron deficiency may be higher for these particular sub-groups compared to their Canadian-born counterparts. This is also likely true for other immigrant groups such as Sub-Saharan Africans. Beneficial effects with regard to fruit and vegetable intakes have also been reported. This is consistent with some studies conducted elsewhere suggesting that the associations between immigration, diet and health is more complex and is not always negative. Indeed, Wallace et al. [15] reported that immigration related acculturation may promote physical activity among US Latinos. Another US study found that acculturation provided a protective effect against type II diabetes among Arab immigrants [44]. Mejean et al. (2009) reported that the impact of acculturation on diet and physical activity among Tunisian migrants living in France was mediated by the past/present exposure to the home country [45]. More research is needed to determine the potential risks associated with dietary acculturation with regards to micro and macronutrient intakes in different immigrant sub-groups.

Findings suggest that many immigrants tend to maintain their traditional diet to a certain extent. Similar results were reported in France where [46] found that immigrants who mostly maintain their Mediterranean pattern of eating tend to experience better nutritional health. Children may lose this health advantage as soon as they acculturate into the new culture [46]. In Canada, such dietary practices present challenges in providing culturally appropriate health services for both immigrants and nutrition professionals. Indeed, nutritional values for ethnic foods are often unknown, which makes the use of Canada’s Food Guide as a reference model for dietary planning difficult in immigrant populations. In addition, nutrition professionals are unfamiliar with ethnic foods and their processing techniques. Immigrant unfamiliarity with grocery stores, lack of awareness of Canadian nutrition discourse and lack of learned cooking skills present additional challenges for immigrants. In attempting to acquire the ingredients necessary for replicating familiar dishes, those immigrants who tend to maintain a traditional diet may be limited to highly processed versions of these foods that are high in fat and sodium; therefore increasing their risk for chronic conditions.

Risk factors for unhealthy dietary habits identified in this review include the availability and accessibility of traditional foods, poverty and financial insecurity, and lack of transportation; all of which are linked with food security. Little is known about the magnitude and coping strategies of food insecurity for Canadian immigrants. Due to unawareness of nutrition discourse, particularly Canada’s Food Guide principles, immigrants tend to derive their nutrition information from sources targeted to their culture including ethnic media and social groups. This, in addition to the factors already mentioned, suggests that culturally appropriate methods for teaching and providing nutrition education to immigrants need to be developed in both the community and health care settings in order to ensure health equity.

Of the studies included in this review, only one was focused on children. In their systematic review of the European literature related to migrant children’s nutritional health, [47] found that immigrant children are at increased risk for overweight (8.9–37.5 % vs. 8.8–27.3 %) and obesity (1.2–15.4 % vs. 0.6–11.6 %) as compared with native children [47]. Childhood obesity is radically increasing in Canada with a subsequent adolescent onset of nutrition related chronic conditions such as type II diabetes. Therefore, children who immigrate to Canada or are of descent from immigrants of South Asian, Latino, Caribbean and South African origin are at increased risk for developing these conditions at an earlier age. Studies geared exclusively to immigrant teenagers and children are needed in order to identify and develop culturally appropriate interventions for this high-risk group. This type of research would also be useful in determining whether children who immigrate to the country at an earlier age are at a higher or lower risk for dietary and health transitions compared to those who immigrate later (i.e. as teenagers) over time.

An important issue raised by Lear et al. [34] is a potential dilemma experienced by immigrants to decide between retaining the values of their home culture and adapting to those in their new country. The number of years since immigration and ethnicity appeared to be poor determinants of the dietary habits and health status of immigrants in some studies. Although some scales of dietary acculturation were developed and applied in the studies reviewed, they were limited in their applicability because they focused on only one specific subgroup and/or were based on broad categorization. A more specific and validated model would allow standardized results from numerous studies to be compared among different cultural and ethnic groups. It is possible that the health and nutrition impact of immigration is better correlated with the extent to which a person has maintained their home country traditional diet or accommodated to the host country lifestyle. This suggests a pressing need for improving acculturation measures in Canada through well designed research studies.

An important limitation of this scoping review is the insufficient focus on mental health of immigrants in relation to their dietary habits, which is an important concern considering the stress associated with a drastic change to one’s cultural surroundings. Indeed, there is rapidly growing evidence in the literature that immigrants disproportionately experience poverty, unemployment, and psychosocial factors such as loneliness and inappropriateness of the social support system in Canada [48–58]. Further, although diet is the focus of this review, there are numerous other immigration-related factors linked with the broader social determinants of health for newcomers to Canada and these factors must not be disregarded. The effects of migration on the health of individuals and groups are vast and multi-dimensional and, as such, require consideration from an interdisciplinary perspective.

## Conclusions

The systematic approach applied in this study enabled a scoping assessment of the current literature related to immigrant acculturation, diet and health in Canada and led to the identification of potential key priorities for research in the area of immigrant nutritional health. The few studies that were available for the review tended to support a steady transition in dietary habits despite some effort to maintain a traditional diet.

Findings suggest that research pertaining to immigrant nutritional health in Canada is embryonic and may evolve quickly as there are important issues that need to be addressed and an urgent need for evidence to guide culturally appropriate practice. Consistently, with the increasing proportion of non-European/non-American immigrants amongst more recent arrivals and emerging interest in immigrant health, increased attention towards research related to nutrition and health transitions among these highly vulnerable sub-populations should be a priority. This review has identified major knowledge gaps that were confirmed by a stakeholder consultation through a survey and a symposium. Further, stakeholders including researchers, service providers (community health centers and NGOs working with immigrants), policymakers, beneficiaries and other relevant partners have identified key priorities in research in a consensual manner. Addressing these priorities would provide relevant data to guide culturally-situated interventions that are suitable for both the Canadian health and nutrition environment realities, and the immigrant’s unique cultural needs, thus reducing the burden of the *healthy immigrant effect* on the health systems.

## Electronic supplementary material

Below is the link to the electronic supplementary material.

Supplementary material 1 (60 kb)

## Footnotes

1 For the search criteria, the * denotes that this term was used as a *root word* so that any words containing that segment would be recognized by the search engine. For example, Canad* = Canada, Canadian and Canadians.
