# Supplementary material for: Acculturation and Nutritional Health of Immigrants in Canada: A Scoping Review
Source: J Immigr Minor Health. 2013 Apr 18;16(1):24–34. doi: 10.1007/s10903-013-9823-7 (PMC3895180; doi:10.1007/s10903-013-9823-7)
Supplement: Supplementary file 1 — Supplementary material 1 (60 kb) [file 10903_2013_9823_MOESM1_ESM.docx]

***Appendix 1: Key features of the 49 papers included in the scoping review***

| **Reference** | **Study Design** | **Study population characteristics (age, setting, ethnic group) and sample size)** | **Acculturation measure** | **Dietary /Health status measure** | **Results** |
| --- | --- | --- | --- | --- | --- |
| Ahmed 2005 | NLSCY Cycle 2 (1996 - 97) | School-aged children (4 - 13 years old) Respondent = Resident Person Most Knowledgeable about child (PMK)  N= 11 617 total 1402 = immigrant families 10 217 = Native-born Canadian families | Time since immigration (YSI) of single-parent (or PMK in families with 2 foreign-born parents) Country of origin = - American - European - Asian | PMK's subjective assessment of child's overall health (poor /fair /good /very good /excellent) | Overall, no substantial difference btw health of children from immigrant and NBC families BUT differences among sub-groups: Compared to NBC; American children = ↑Health Status; Asian children = ↓HS, Euro children = same HS. Therefore HS-Asian /Euro is probably worse than NBC (if American children are not considered) Assimilation models: ↑parent's time of residency in Canada = ↑child health outcome (match to NBC in 3-4 years) |
| Cairney & Ostbye; 1999 | NPHS | Adults; aged 20 - 64 Pregnant women excluded  N = 11 818 | Time since immigration, 4 categories: Born in Canada  0-4 YSI; 5-9 YSI; 10+ YSI Country of origin | BMI (self-reported weight and height) | Women: ↑ BMI with ↑ YSI (after 10 years, immigrant = higher prevalence of overweight than native-born females) Men: ↑ BMI with ↑ YSI (Asian origin only) Not attributable to other demographic, lifestyle and health factors |
| Cervellon & Dubé; 2004 | FFQ - modified (based on food preferences) | Adults of Chinese origin living in Canada, France and China  N = 329 118 France 100 PR Chinese 111 Chinese-Canadians | Time since immigration   Language proficiency (participants selected if able to fill out the questionnaire in English) | Most liked and disliked foods  Reasons given for preferences: *Affective* (sensorial, emotional, social) vs. *Cognitive* (health, convenience, function) | Food: Chinese-Canadians = ↓ Grains (rice), ↓Dairy and ↑ Veg compared to PR Chinese Likes: Both Chinese-Canadians and PR Chinese = balance between affective and cognitive basis Dislikes: PR Chinese = balance between affective-cognitive basis; Chinese-Canadians = strongly AFFECTIVE basis Demonstrates resistance to acculturation and food aversions developed due to exposure to unfamiliar foods in a new culture |
| Chen et al; 1996 |  | Adults - aged 18 +  N= 41 045 (Can.Born = 34666, Euro.Imm = 4004, Non.Euro.Imm = 2375)For estimates of behavioural risk factors: N = 16 291 (CB = 13 947, EI = 1640, NEI = 704) | Time since immigration, 2 categories: Recent ≤ 10 years, Long-Term > 10 years | Presence of chronic conditions (disability, health-related dependency, health-care utilization)Health-related behavioural risk factors (smoking, physical activity) | Recent immigrants (esp. NEI) = ↑health compared to Canadian-born population> 10 years YSI = ↑ chronic conditions, ↑ long-term disability↑ time in Canada = ↑ similarity in health-related lifestyle behaviours to Canadian-born population |
| Cleveland et al; 2009 | FFQ  Ethnic identity questionnaire | Adult Lebanese-Canadians (LC) residing in Montréal area  N = 166 | Time since immigration  Country of origin Religious affiliation Language use Level of social interaction  Ethnic identification Family structure and gender roles Desire to maintain Lebanese culture | Frequency of consumption Lebanese-traditional versus French-Canadian foods; comparison of foods with similar composition (i.e. Kibbi vs. hamburger steak) | The relationship between acculturation and food consumption is more complex than simply ↑ ethnic identity = ↑ traditional food consumption and ↑ acculturation = ↑ mainstream food Integration model = blending of influences from home and host cultures Level of ethnic identity or acculturation in terms of consumption = dependent of food category |
| Dean & Wilson; 2010 | Semi-structured interviews | Adult Immigrants residing in the Greater Toronto Area (GTA)  N = 23 | Time since immigration, 3 categories: recent < 3 years mid-term 3 - 10 years long-term > 10 years | Self-assessed health status - SAH (VG, good, fair, poor, VP) Perceived change in health status Changes in health care usage, eating habits, physical activity and tobacco /alcohol consumption | Only 7 in 23 reported worsening of health; distributed evenly among YSI categories (does not support *healthy immigrant effect*) Access to healthcare = overall negative view Eating habits = mixed - better access to healthy foods but also ↑ opportunity to get unhealthy foods Phys activity = mixed - safer but more $$ Tobacco /alcohol = ↓ usage overall compared to pre-immigration |
| Delisle; 2010 | FFQ - semiquantitaive (Madrid)24 hr recalls: 2 or 3 non-consecutive (Montréal) | Adults with no prior diagnosis of diabetes or CV disease  N = 935 (181 Montréal Haitians, 213 Madrid Bubi, 541 West Africa Beninese) | Immigrant status only (living in Montréal and Madrid) | *Healthfulness score* of diet = compliance with WHO guidelines for prevention of chronic diseasePositive = F & V, fish, whole grains and legumesNegative = sweets, processed foods, fried foods, fats and oils, salty snacks | Traditional diets = more positive (healthful); Western-type diets = more negative (unhealthy)Dietary transition model - Identification of 4 patterns: T*raditional, Pre-Western, Western* and *Modern* (most common among Montréal Haitians = Traditional and Pre-Western) |
| Désilets et al; 2006 | 24 hour recalls: 3 non-consecutive  Questionnaire | Adult Haitian immigrants living in Montréal; aged 25 - 60  N = 181 | Proportion of lifetime in Canada: 0 - 44% 45 - 54% 55 - 100% | Level of dietary transition: *Traditional* → *Pre-Western* → *Western*→ *Modern* Healthfulness of diet = based on compliance with WHO guidelines for prevention of chronic disease and micronutrient intake | Majority of subjects = *Traditional* or *Pre-Western* (83 % together) Maintenance of *Traditional* dietary pattern associated with ↓ proportion of life in Canada, ↑age and ↓socioeconomic status  *Traditional* diet (↑ F & V, low fat dairy) = significantly more healthful than *Western* diet (↑ processed foods) |
| Dhaliwal; 2002 | Semi-structured interviews  Observations in participant's homes | Older-adult Punjabi women; aged 52 - 64 Residing in West Toronto with family (5 - 15 years)  N = 9 | Time since immigration  Cultural knowledge, norms, beliefs and practices | Health related eating behaviour, adherence with 4 themes: '*Body knows its needs' 'Tasty = healthy' 'Tradition drives practice' 'Change in environment. = change in eating habits'* | Effort to maintain traditional practices, but moving to Canada = some changes in eating habits Changes mainly due to different living environment, availability of foods, and lifestyle factors (urbanization, language barriers) |
| Dunn & Dyck; 2000 | NPHS (1994 -95) | Adults - aged 20 +  N = 15 779  (2297 immigrants and 13 482 Canadian-born) | Immigrant status  Time since immigration, 2 categories: Recent ≤ 10 years, Long-Term > 10 years  Country of origin: Euro (US, AUS, Europe) vs. non-Euro (Asia, Africa, S.Amer) | Self-assessed health status (SAH)  Presence of chronic conditions  Healthcare usage | Socio-economic factors = more impact on health status and health care usage for immigrants than for non-immigrants  Disadvantageous SE factors for health status: - immigrants > non-immigrants - non-Euro immigrants > Euro immigrants Euro immigrants = more likely to report poor health, chronic conditions and hospitalization compared to non-Euro immigrants Long-term immigrants more likely to report poor health, chronic conditions compared to short-term |
| Farrales & Chapman; 1998 | Semi-structured interviews | Adult women aged 19 - 30Born in the Philippines to parents of Filipino heritage Living in Vancouver area  N = 11 | Time since immigrationPreferences for language,food, and entertainment | Experiences with food, eating, body image, and healthAdherence to belief system:- Canadian = ↑ thinness, careful with intake of fat, rice and junk food, minimizing disease risk- Filipino = ↑ fatness, unrestricted fat and rice, maximizing disease resistance | Most engaged at least sometimes in *Canadian* health /diet behaviours:- joining a gym- eating sandwiches at lunch- trimming fat from meat- going on a commercial weight loss dietDisplayed mechanisms to bridge somewhat conflicting cultures (i.e. altering traditional Filipino dishes to reduce fat content) |
| Gee et al; 2004 | CCHS (2000-01) | Adults of mid-life age (> 45 years)  N = 54 848 45 - 65 yrs of age: Can Born = 27695 Imm>10 yr = 7634 Imm<10 yr = 1206  65 + yrs of age: Can Born = 13488 Imm>10 yr = 4525 Imm<10 yr = 300 | Immigrant status  Time since immigration:  Long-term > 10 YSI Recent < 10 YSI | Self-assessed health status (SAH) - Positive = good, VG or excellent - Negative = fair or poor  Global health status measures: - Healthy Utility Index (HUI) - Activity Restriction (AR) | The healthy immigrant effect applies to later mid-life immigrants Recent mid-life (45–64 years) immigrants = better SAH and AR compared to long-term  For ages 65+, recent immigrants have poorer overall health compared to long-term and Canadian-born (this disadvantage disappears when sociodemographic and socio-economic factors are held constant) |
| Hyman & Dussault; 2000 | Semi-structured interviews | Adult pregnant women from South-East Asia (Vietnam, Cambodia or Laos) residing in Montréal QC  N = 17 | Time since immigration - 2 waves:  1975 (more accultured) 1978 (less accultured)  Language proficiency (English and /or French) | Health behaviours (diet, smoking, alcohol consumption)  Level of social support  Stress | Acculturation = negative health consequences for immigrant women ↑ Time since migration = ↑ preoccupation with thinness and dieting during pregnancy More recent immigrants = better social support network Long-term immigrants = more stress, pressure to adhere to host- country cultural norms |
| Johnson & Garcia; 2003 | 24 hour recall  Questionnaire (background)  Nutritional risk assessment  Physical activity assessment | Elderly adult immigrants aged 59 – 81; Residing in London Ontario  N = 54  Cambodian = 11 Latin-American = 15 Vietnamese = 13 Polish = 15 | Immigrant status  Language proficiency (English and /or French) | Adherence to Canadian Dietary Guidelines (CDG) | Most participants did not speak, read, write, or comprehend English = low level of acculturation Most = moderate to high risk of poor nutrition; attributed in part to the continuance of traditional eating habits and consumption of special ethnic foods Higher carbohydrate and lower fat intakes compared to CDG Excessive sodium intakes (238 to 474% compared to CDG) - salty condiments, pickled vegetables, and salted fish |
| Kaplan et al; 2003 | NPHS (1996-97) | Adult Asian immigrants; aged 20+  N = 1972 | Time since immigration (YSI), 3 categories:0 - 4 years5 - 9 years10 + years | Diagnosis of high blood pressure by a health care professional | ↑ Prevalence of hypertension (HT) follows degree of cultural adaptation (↑ YSI)May be result of lifestyle changes and dietary practices (meal patterns and food choices) |
| Kopec et al; 2001 | NPHS (1994-95) | Canadians aged 12+  N = 15 960 | Country of origin  Language proficiency (English and /or French) | Health Utilities Index (based on eight attributes: vision, hearing, speech, mobility, dexterity, cognition, emotion, and pain/discomfort) Presence of chronic conditions | Bilingual Canadians and Eng-speaking immigrants = ↓ classification as dysfunctional compared to English Canadians  Bilingual Canadians + non-English speaking Euro and Asian immigrants = ↓ healthy category compared to Eng Canadians |
| Kwok et al; 2009 | FFQ  Questionnaire | Older-adult Chinese-Canadians (born in China, Taiwan or Hong Kong); aged 45 - 64  Residing in Toronto  Excluded = those with diet restrictions due to chronic illness  N = 106 | Time since immigration  Age at migration  Adherence to Traditional Health Beliefs (THB-weak, THB-moderate and THB-strong) | Dietary behaviours: - Fat-related behaviours - Fruit and vegetable consumption | Dietary acculturation = fusion of the two cultures Younger age at migration = ↓ THB-strong;  > 10 yrs residency = doubtful of THB Recent immigrants rarely ate Western foods THB-strong = ↓ reduction of added fats and oil in cooking THB-weak = ↑ trimming of fat off meats Traditional Chinese F & V (Chinese turnips, bitter melon) were more frequently consumed than Western varieties (lettuce salads, fruit juice, potatoes) |
| Laroche et al; 2005 | FFQ   Questionnaire | Adult Italian-Canadians and Greek-Canadians; aged 20+ Residing in a major metropolitan area of Eastern Canada  N = 1000 (500 each Italian-Canadians and Greek-Canadians) | Ethnic language use with family members  Ethnic-language media exposure  Ethnic attachment | Consumption frequencies for traditional and convenience food products | For both Ital-Can and Greek-Can groups, ↑ ethnic identity = ↑ consumption of traditional foods Ethnic identity = negatively related to consumption of convenience foods for Ital-Can group only Acculturation = no effect on traditional and /or convenience food consumption for either group |
| Lear et al; 2009 | Food record (3-day)Questionnaire | Adult Canadians with Chinese, European or South Asian ethnicityMinimum 3 years residency in Canada Maximum 4th generation CanadianFree of previous CVD diagnosis or co-morbidity  N = 618 (460 immigrants and 158 non-immigrants) | Time since immigration (YSI), 4 categories:< 10 years10 - 20 years20 - 30 years30 + yearsCountry of origin | Risk of CVD including:BMI and anthropometric measuresDiet = Caloric, dietary fat and saturated fat intakeExercise and lifestyle factorsOverall Self-assessed health status (SAH) | Time since immigration was positively (but weakly) correlated with age, dietary fat intake, physical activity and lipid measures↑ risk of CVD with time since immigration, but underlying reasons are uncertain |
| Lu et al; 2008 | Semi-structured interviews | Adult Chinese-Canadians - First generation; aged 25 - 50  Canadian permanent resident or citizen Residing in a large city in Western Canada  N = 10 | Immigrant status - born in China | Changes in food choice, meal style and cooking methods since immigration | Preference for Chinese foods with adoption of some Western foods (i.e. breakfast) Combined Chinese cooking with Western prepared meat (e.g., sausages), canned foods (e.g., beans, tomato sauce, corn), and frozen vegetables to create a hybrid type of food Occasionally ate “junk food” even when aware of the negative health consequences; usually to please younger family members |
| Marquis & Shatenstein; 2005 | Food Choice Questionnaire (FCQ) | Mothers of school-aged children  Country of origin = Haiti, Portugal or Vietnam Residing in the Montréal area  N = 209 (68 Haitian, 75 Portuguese, 66 Vietnamese) | Time since immigration  Country of origin  Religious affiliation | Food choice motives Importance attributed to family meals | Significant difference of food choice motives based on country of origin Five major factors accounted for most food choice motives (convenience, health, pleasure, familiarity and ingredient properties)  Haitian & Portuguese = health Vietnamese = health + familiarity of foods  Health & pleasure → overall predictor of family meal importance |
| McDonald & Kennedy; 2004 | NPHS (1996) CCHS (2000-01) | Adult aged 20 - 65  N = 139 931 (18 754 born outside of Canada; 121 177 born in Canada) | Time since immigration (YSI)  Country of origin ESB = UK, USA, Euro, Aus & NZ NESB = continental-Euro & Asia | Self-assessed health status (SAH) - excellent, VG, good, fair or poor  Presence of chronic conditions  Use of healthcare services | Confirmation of the healthy immigrant effect (recent immigrants = ↓ chronic conditions and; difference narrows with YSI) Only weak evidence of change in SAH  Cohort /period of arrival = statistically significant determinant of immigrant health  After 20 - 25 years = convergence to native-born levels, but does not worsen past there Only certain conditions (i.e. asthma, allergies, HT, migraines, ulcers, arthritis) = ↑↑ for NESB compared to ESB recent immigrants |
| McDonald & Kennedy 2005 | NPHS (1996)CCHS (2000-01)Canadian Census Files (1996, 2001) | Adults aged 21 - 65Excluded = First Nations peoples and those reporting to belong to multiple or unspecified ethnic groups  N = 126 796 | Time since immigration (YSI)Visible minority status Mother tongueCensus subdivision (CSD) (ethnic group concentration of surroundings) | BMI (self-reported weight and height) | *Healthy immigrant effect* varies by ethnicity↑ excess weight with YSM is tempered by the presence of significant ethnic social network effects: if an individual resides in a neighborhood with a relatively large ethnic community and the ethnic group is less likely to be overweight or obese than the average Canadian, then the individual is also less likely to be overweight or obese |
| McDonald; 2006 | NPHS (1996) CCHS (2001-02 and 2002-03) | Adults aged 20 - 65  N = approximately 130 000 | Time since immigration (YSI), 3 categories: < 10 years 11-20 years 21 + years  Country of origin: ESB (from English speaking country), NESB-Europe, NESB-other  Race: White vs. visible minority | Health related behaviours: 1- weekly drinker 2- binged on alcohol (5+ drinks) in the last month 3- daily smoker 4- engaged in physical activity in the last week 5- typically consume 5+ servings of F or V per day | Immigrant groups = ↓alcohol ↓tobacco, but also ↓physical activity and F/V consumption (esp. NESB-other) Some evidence of convergence to Canadian-born over time for men (esp. Smoking and alcohol consumption) ESB and NESB-Euro = reach native-born levels in 10 -20 years NESB-other = alcohol consumption remains low No change in health behaviours with time for women |
| Newbold & Danforth; 2003 | NPHS (1998-99) | Canadians aged 12 +  Excluded = First Nations peoples and individuals residing in institutions  N = 17 244 (2058 immigrants and 15 186 Canadian-born) | Time since immigration (YSI), 3 categories: 0 - 4 years 5 - 9 years 10 + years  Country of origin (America, Europe or other)  Race (White or other) | Self-assessed health status (SAH) - excellent, VG, good, fair or poor  Presence of chronic conditions  Health Utilities Index Mark 3 (HUI3) | Immigrants= ↑poor SAH and ↓ reporting better states of health Near continuous decline in health status, (HUI3 or SAH) for immigrants with ↑ YSI; decline towards Canadian-born levels in first 10 years regardless of age Chronic conditions: recent immigrants < Canadian-born < immigrants in Canada 10+ years (esp. diabetes, heart disease, and arthritis) American and Euro immigrants report ↑rates for chronic conditions - asthma and heart disease (but age may be a factor) |
| Newbold & Filice; 2006 | CCHS (2000-01) | Older adults aged 55 +  N = 38 474 (6780 immigrants and 31694 Canadian-born) | Immigrant status | Self-assessed health status (SAH) - excellent, VG, good, fair or poor Presence of chronic conditions Health Utilities Index Mark 3 (HUI3) | Immigrants rank similarly to native-born Canadians in SAH, HUI3 and number of chronic conditions  Immigrants may be lower in HUI3 score, depending on type of analysis |
| Newbold; 2009 | LSIC | Immigrants to Canada; aged ≥ 15 years old at time of arrival Arrival between Oct 1, 2000 and Sept 30, 20013 categories: Economic, Family and Refugee  N = 7700 | Time since immigration (YSI), 3 waves:1) 6 mths2) 2 yrs3) 4 yrs | Self-assessed health status (SAH)*Healthy* = good, very good or excellent*Unhealthy* = fair or poorEmergent physical or mental health problems | Rapid decline in SAH after two years YSIAfter 4 years: approx 2x fewer immigrants report *excellent* health, 3x more report *poor* healthDeclining SAH corresponds to ↑ in new mental and physical health problemsImmigrant class = significantly correlated to health status (Refugees = 2x more likely to report *poor health* compared to family or economic class immigrants)  Sociodemographic factors = dominant contributor to declining health status (age, gender, origin and immigrant class) |
| Newbold; 2009 | NPHS - 4 cycles (1994-95,  1996-97,  1998-99, 2000-01) | Adults aged 20 +  N = 17 276 (1305 immigrants and 15 971 Canadian-born) | Immigrant status  Arrival cohort:  - 1990–1994 - 1980–1989 - 1970–1979 - pre-1970 | Incidence of health care use: - - Visits to general practitioner (contact with GP within the past year) - Hospital use (overnight stay within the past year) | Inconsistent with *healthy immigrant effect* Relative equality of health care use by foreign and native born populations Little evidence of increased health care use with earlier arrival cohorts Hospital use /frequency and GP visits = same for immigrants and native-born even with concomitant declines in self-assessed health for immigrants |
| Newbold; 2006 | NPHS - 4 cycles (1994-95,  1996-97,  1998-99, 2000-01) | Foreign-born adults aged 35+ in 1994-95 survey  N = 911 | Immigrant status  Arrival cohort:  - 1990–1994 - 1980–1989 - 1970–1979 - pre-1970 | Chronic conditions (CC): Presence Number Type (CVD, arthritis, asthma, diabetes) | Support of the healthy immigrant effect with respect to chronic conditions (recent arrivals = lower levels of CC than those arriving earlier); this advantage ↓ with time - converging to native-born level |
| Newbold; 2005 | NPHS - 7 cycles (1994/95 - 2000/01) | Adult immigrants to Canada; aged 20+  N = 1305 | Immigrant status  Arrival cohort:  - 1990–1994 - 1980–1989 - 1970–1979 - pre-1970 | Changes in self-assessed health status (SAH)  Presence of chronic conditions  Incidence of health care use  Relative risk of transitioning from *healthy* to *unhealthy* SAH over time compared to native-born population | Consistent with *healthy immigrant effect* Most recent cohort (1990-94) = most drastic decline in SAH and increase in chronic conditions (despite younger average age) Percentage reporting chronic conditions increased in other cohorts as well, but not to the same extent Visits to GP in past year ↑ for each cohort over time, but hospital usage did not↑ Increased use of health care with YSI can not be attributed to increase in age alone Overall, no ↑ or ↓ risk of declining health for immigrants compared to native-born (in analysis including socioeconomic, demographic and lifestyle factors) |
| Newbold; 2004 | NPHS - 4 cycles (1994-95, 1996-97, 1998-99, 2000-01) | Adults aged 20 +  N = 17 276 (1305 immigrants and 15 971 Canadian-born) | Immigrant status Arrival cohort: - 1990–1994- 1980–1989- 1970–1979- pre-1970 | Self-assessed health status (SAH)*Healthy* = good, very good or excellent*Unhealthy* = fair or poor | Mixed support for the *healthy immigrant effect*At a given time, foreign-born were not more or less likely to rank their health as poor relative to the native-born populationOver time, native-born were at a lower risk of transitioning to poor health than immigrantsPre-1970 arrivals = much less likely to transition from a *healthy* to an *unhealthy* state compared to later-arrivals (recent arrivals experienced particularly dramatic declines in SAH) |
| Ng et al; 2005 | NPHS - 5 cycles (1994/95 - 2002/03) | Adult immigrants to Canada; aged 18 +  14 117 total:  (12 038 Canadian-born, 2079 immigrants) | Time since immigration (YSI) - Recent (≤ 1984) - Long-term (>1984)  Country of origin - European (US, AUS, NZ, Europe) - Non-European (all other countries) | Changes in self-assessed health status (SAH): transition from good /very good /excellent to fair or poor;   Frequency of physician consultations (> 6 /year)  Relative risk of BMI increase by 10% or more | Only non-European immigrants = significant increase in relative risk for reporting transition Recent non-Euro immigrants = significant relative risk of 10% or more gain in BMI and doctor contacts > 6 /year Although negative lifestyle factors (inactivity and smoking) were significantly higher for Euro immigrants, non-Euro immigrants were more likely to report health deterioration, more frequent doctor's visits and weight gain |
| Oliffe et al; 2010 | Semi-structured interviews  Observations at mens-group meetings | Older-adult Punjabi-Sikh men, aged 63 - 88 Residing in British Columbia Living in Canada 9 - 41 years  N = 35 | Alignment with masculine ideals rooted in Punjabi-Sikh (PS) culture (attachment to spiritual and traditional practices) | Adherence to PS culture or Western norms with regards to diet | Overall preference for traditional dishes but availability and affordability of food /beverage in Canada = influence on diet |
| O'Loughlin et al; 2007 | FFQ   Questionnaire (self-administered) | Parents of elementary school children (grades 4 - 6) from 24 school in Montréal: 8 Schools in St. Louis du parc (multi-ethnic, economically disadvantaged neighbourhood) 16 comparison schools (all = in lowest 25% on poverty index)  N = 2033 | Family origin = based on: - Country of birth - First language learned in childhood | Lifestyle risk factors: 1- Smoking 2- Physical inactivity 3- BMI 4 - Dietary habits (*Junk food consumption score* and *Fruit and Vegetable consumption score*) | Co-occurrence of lifestyle risk factors = highest for French Canadians (host culture) Ethnic groups with highest proportion of participants born in Canada (Italian, Portuguese) = ↑ co-occurrence of lifestyle risk factors (suggests ↑ YSI = ↑ lifestyle related risk factors) Asian family origin = most favourable risk profile (except South Asians)  Considerable variability of risk factors across groups |
| Perez; 2002 | CCHS (2000-01) | Canadians aged 12 +Excluded = First Nations peoples and individuals residing in institutions  N = 131 535 (16901 immigrants and 114 634 Canadian-born) | Time since immigration (YSI): - 0-4 years- 5-9 years- 10-14 years- 15-19 years- 20-29 years- 30+ yrs | Incidence of chronic conditions:-General-Heart disease-Diabetes-High blood pressure-Cancer | Immigrants = lower rates of reporting chronic disease, but rate ↑ with ↑ YSIHeart disease: only recent immigrant men = healthier than non-immigrantsCancer: only recent immigrant women = healthier than non-immigrantsAll other conditions, immigrants = same rates as non-immigrants |
| Pillarella et al; 2007 | Semi-structured interviews  Questionnaire | Francophone West-African immigrants; aged 20 - 43  Residing in Montréal ≥ 10 yrs  Person responsible for household groceries and food preparation  N = 10 (4 women, 6 men) | Placement on an *Acculturation Scale*; assimilation, integration, separation and marginalization (based on cultural identification and loyalty) | Maintenance of African dietary habits vs. adoption of Canadian dietary habits  Identification of the reasons for making changes since immigration  Understanding of link between diet and health  Knowledge and /or interest of Canadian food habits | Inevitable change in dietary habits with migration but remain mostly African-based, independently of time since migration  Knowledge and interest of Canadian dietary culture = limited  Proposed schema of dietary acculturation: Assimilation (1-3 mths) →Adaptation Phase →Ethnocentrism →Open Ethnocentrism →Integration Most changes due to time constraints, unfamiliarity with grocery stores, new interpersonal relationships Reasons for maintenance of traditional dietary habits: learned cooking skills, taste preferences, health knowledge |
| Pomerleau & Østbye; 1997 | Ontario Health Survey (1990) | Adults aged 18 + Residing in Ontario  N = 43 292  (8848 immigrants and 34444 Canadian-born) | Region of origin  Reported ethnicity -Canadian -Canadian + other -Other but not Canadian | Rates of obesity and overweight  Health-related lifestyle behaviours (smoking, drinking, diet, physical activity)  Reports of chronic conditions | Immigrants = ↓ obesity compared to native-born (esp. Asian group) and ↓ reports of chronic conditions Immigrants = ↓ smoking, drinking and fat consumption compared to native-born, but also ↓ physical activity |
| Pomerleau & Østbye; 1998 | FFQ (developed for OHS)  Ontario Health Survey (1990) | Adults aged 18 + Residing in Ontario  N = 36 616 (7158 immigrants and 29458 Canadian-born) | Region of origin -Canadian -Other | Frequency of higher or lower intakes than recommended of energy, fat, cholesterol, carbohydrate, fibre and alcohol compared to Canadian Recommended Nutrient Intakes | Immigrants = less likely to consume more fat, less carbohydrate and more alcohol than recommended compared to native-born population Cholesterol and fibre intakes were not strongly associated with place of birth |
| Pomerleau et al; 1998 | FFQ (developed for OHS)Ontario Health Survey (1990) | Adults aged 18 +Residing in Ontario  N = 36 616 (7158 immigrants and 29458 Canadian-born) | Region of origin-Canadian-Other | Protein, calcium, iron, vitamin C, thiamine, riboflavin, and niacin intakes compared to Canadian Recommended Nutrient Intakes (compared to WHO recommendations for calcium only) | Immigrants (esp. from Asian countries) = higher risk of inadequate intakes of protein, calcium and iron compared with non-immigrants |
| Pottie et al; 2008 | LSIC - 2 cycles (2001, 2003) | Canadian immigrants; aged 15 +  Arrival to Canada between Oct 2000 and Sept 2001  Excluded = Immigrants claiming refugee status  N = 11 802 | Time since immigration (YSI), 2 waves: 1) 6 mths 2) 2 yrs  Language proficiency in English and French (French in Québec only) - Speaking abilities = "Good" or "Poor" | Self-assessed health status (SAH) *Good Health* = excellent, very good or good *Poor Health* = fair or poor | Poor proficiency in English or French = significantly related to poor SAH for women (but not men) at 6 mths and 2 years post-immigration |
| Saposnik et al; 2010 | PRESARIO - Risk of Premature Stroke Associated with Recency of Immigration to Ontario  (matched retrospective cohort study) | Adults aged 16 - 65  Residing in Ontario  N = 4 238 222 (965 829 new immigrants; 3 272 393 long-term residents) | Recency of immigration: New immigrants = received OHIP number between Apr. 1995 and Mar. 2006 Long-term residents = valid OHIP number 5+ years between Apr. 1995 and Mar. 2006 | Hospitalization with diagnosis of acute premature stroke | New immigrants appear to be at lower risk of premature acute stroke than long-term residents |
| Saposnik et al; 2010 | MARIO - Myocardial infarction Associated with Recency of Immigration to Ontario  (matched retrospective cohort study) | Adults aged 16 - 65  Residing in Ontario  N = 4 238 222 (965 829 new immigrants; 3 272 393 long-term residents) | Recency of immigration: New immigrants = received OHIP number between Apr. 1995 and Mar. 2006 Long-term residents = valid OHIP number 5+ years between Apr. 1995 and Mar. 2006 | Hospitalization with diagnosis of acute myocardial infarction (AMI) | New immigrants had 35% lower relative risk of AMI than matched long-term residents |
| Satia et al; 2001 | FFQ  Questionnaire  Interview | Adult women; aged 20 +  Chinese ethnicity  Residing in Vancouver BC (131) and Seattle WA (113)  N = 244 | Age at immigration ≤ 25 years old 26 - 39 years old > 39 years old  Media preferences (newspaper /magazine, music, television) | Fruit and vegetable intake  Fat-related behaviour  Dietary change with regards to fruit, vegetable and fat intake | 2 scales established: Western and Chinese ↓ age, ↑ education and ↑ proportion of life in N. America = highest Western dietary acculturation scores High scores on Western scale = higher fat and increased fruit and vegetable consumption since immigration |
| Satia; 2000 | QuestionnaireHousehold food inventory | Adult women; aged 20 +Chinese ethnicityResiding in Vancouver BC (131) and Seattle WA (113)  N = 244 | *Chinese /American Dietary Acculturation Scale Score*: westernizationof dietary patterns (snacking between meals, eating at fast-food restaurants,and eating dairy products)Proportion of time in North America | Presence of 14 specified high-fat and 7 reduced-fat food items in the householdFat-related dietary behaviourChanges in consumption of high-fat foods | Less-accultured sample, but many households had Western foods such as butter, lunchmeats, snack chips and milk↑proportion of life in North America = ↑ number of reduced-fat foods in householdFat intake increases with Western acculturation and affluenceWestern dietary acculturation correlatedpositively with having both more high- and reduced-fat foods |
| Satia-Abouta et al; 2002 | Questionnaire | Adult women; aged 20 +  Chinese ethnicity  Residing in Vancouver BC (131) and Seattle WA (113)  N = 244 | *Chinese /American Dietary Acculturation Scale Score*: westernization of dietary patterns (snacking between meals, eating at fast-food restaurants, and eating dairy products)  Proportion of time in North America | Consumption of foods associated with Chinese and Western cultures  Pre- and post-immigration consumption of fruits, vegetables and fat | Demographics = associated with psychosocial factors related to diet; psychosocial factors modestly predicted adoption of Western dietary patterns ↑ age ↓ education = more 'Chinese' diet (low fat, high fruit and vegetable intake) ↓ age ↑ education = did not consider Chinese diet as healthier than Western diet Respondents with in-family normative pressure tended to maintain Chinese eating patterns and ate more fruits and vegetables |
| Setia et al; 2009 | NPHS - 7 cycles (1994 -2006) | Adults aged 18 - 54 (at baseline survey)  N = 5466 (587 immigrants and 4879 Canadian born) | Time since immigration (YSI)  Race; white immigrants (WI) and non-white immigrants (NWI) | BMI - calculated from self-reported height and weight | Only male WI showed a positive mean change in BMI over 12 years Even after adjusting for time since immigration, non-white immigrants had lower BMI than white immigrants Not all immigrants experience convergence of BMI to Canadian native values |
| Tremblay et al; 2005 | CCHS - 2 cycles (2000/01, 2003) | Adults aged 20 - 64  Excluded = First Nations peoples and individuals living on Canadian Forces bases and in some remote areas  N = 84 709 | Time since immigration (YSI):  ≤ 10 years (recent) > 11 years (long-term)  Ethnicity (self-declared cultural and racial background) | BMI - calculated from self-reported height and weight | BMI differs significantly by ethnicity Overweight /obesity: Off-reserve Aboriginals > Whites > East /Southeast Asians Prevalence of overweight /obesity = ↑ for long-term compared to recent immigrants Long-term immigrant BMI = ↑ that native pop. in most cases (Whites, Blacks, Latin Americans, South and West Asians) *Healthy immigrant effect* fades within a decade for all immigrant groups |
| Varghese & Moore-Orr; 2002 | FFQFocus group discussionInterviews | Indian immigrants to Canada (born in India or parents were born India); aged 10 - 65 Residing in Newfoundland  N = 132 | Time since immigration (YSI) | Level of adherence to Canadian Food Guide for Healthy Eating (CFGHE)  Frequency of food choices that are traditional Indian vs. Canadian  Lifestyle practices related to chronic illness  Food choice motives and interest in nutrition issues | Some traditional cooking /eating practices maintained, some abandoned↑ Canadian foods at breakfast, lunch and snacks↑ Traditional Indian foods at supper  Increase in consumption of meat, soft drinks, alcohol, convenience foods, deep-fried foods and butter /margarine spreads |
| Wong & Wong; 2003 | NPHS (1996-97) | Immigrant women; aged 20+  N = 6024 | Time since immigration (YSI)  Country of origin  Conversational languages | Modifiable CVD risk factors (obesity, smoking, physical activity, diabetes, high blood pressure) | Immigrant women = ↑ CVD risk factors compared to non-immigrant women, esp. white race (from USA, Euro and Aus) CVD risk profile of immigrants from USA, Aus, Euro = comparable to Canadian women |
